# Spesolimab successfully treated acute generalized exanthematous pustulosis in an IL36RN-postive patient

**DOI:** 10.1016/j.jdcr.2026.03.056

**Published:** 2026-04-09

**Authors:** Yingkai Tao, Rui Chen, Qingli Gong, Nana Zheng, Yanxia Yuan, Minmin Sheng

**Affiliations:** a Department of Dermatology, The Third Affiliated Hospital of Soochow University, Changzhou, China; b Department of Oncology, The Third Affiliated Hospital of Soochow University, Changzhou, China; c Department of Dermatology, The Second Affiliated Hospital of Nanjing Medical University, Nanjing, China

**Keywords:** acute generalized exanthematous pustulosis, generalized pustular psoriasis, IL-36 receptor antagonist, IL-36RN gene

Acute generalized exanthematous pustulosis (AGEP) is a rare and severe type of drug eruption characterized by acute, extensive, pinhead-sized sterile pustules, erythema, edema, and fever.^1^ Differentiating AGEP from generalized pustular psoriasis (GPP) is challenging. Interleukin (IL) 36 signaling pathway dysregulation is involved in both pustular diseases. Here, we report a case in which IL36RN-positive AGEP was successfully treated with spesolimab [an IL-36 receptor antagonist (RN)] and discuss the possible therapeutic mechanism in AGEP.

## Report of a case

**Fig 1:**
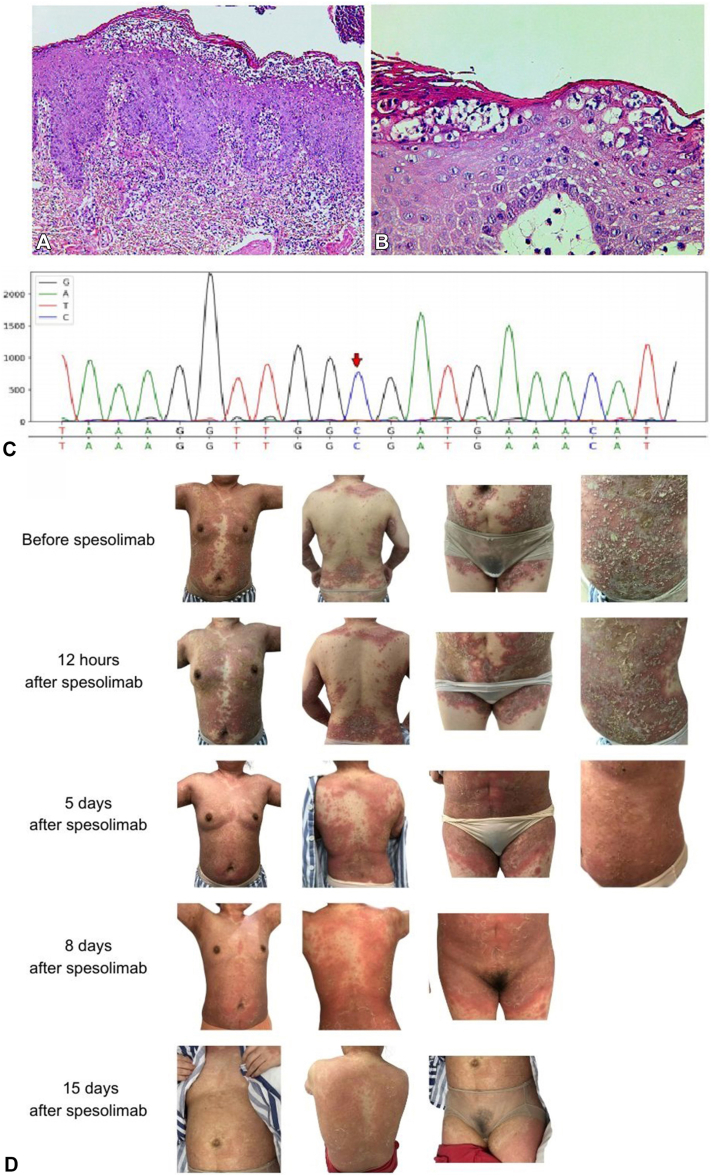
Histopathology, gene map, and clinical features. **(A**, **B)** Histopathology; **(C)** gene IL-36RN pathogenic mutation sites (c.115+6T>C); **(D)** clinical features before and after treatment. (Hematoxylin-eosin stain: original magnifications: **A,** ×40; **B,** ×100)

A 48-year-old woman presented with an acute onset of erythematous and dense pustules throughout her body for 1 week. Two months earlier, the patient had received oral roxithromycin and traditional Chinese medicine to treat neck eczema. No personal or family history of psoriasis vulgaris or allergic history was reported. Dermatologic examination revealed widespread erythematous plaques and dense pustular lesions dispersed across the face, neck, trunk, and limbs. Blood investigations revealed elevated C-reactive protein (18.1 mg/L; normal range: 0-10 mg/L) and liver enzyme levels (alanine transaminase: 117.1, 7-50 U/L; aspartate transaminase: 39.4, 13-40 U/L; ALP: 129, 35-100 U/L; and gamma-glutamyl transferase: 227.9, 7-60 U/L). The results of bacterial culture and fungal tests were negative. Dermatopathologic findings revealed Munro microabscesses, subcorneal pustulosis, dermal spongiosis, and a superficial perivascular lymphocytic infiltrate admixed with some eosinophils and neutrophils (Fig 1, *A* and *B*).

AGEP or GPP was suspected. Intravenous methylprednisolone (0.75 mg/kg/d) was administered for 8 days. However, symptoms continued to worsen, with more erythema and pustules appearing on the trunk, accompanied by swelling in the limbs, fatigue, and fever (37.7 °C).

A genetic analysis was performed. The pathogenic homozygous mutation (c.115+6T>C) in intron 3 of the IL36RN gene was identified (Fig 1, *C*).

As our patient may have had IL-36 dysfunction, she was treated with 900 mg of spesolimab. After 12 hours, areas of pustules on her trunk had dried. The swelling in her limbs was relieved, and her body temperature returned to normal. After 5 days, the pustules completely disappeared. Notably, the levels of C-reactive protein and liver enzymes significantly decreased (C-reactive protein: 7.6 mg/L; alanine transaminase: 56.9 U/L; aspartate transaminase: 21.4 U/L; ALP: 101 U/L; and gamma-glutamyl transferase: 185.8 U/L), indicating that the inflammatory cascade was effectively controlled. After 15 days, the erythema completely subsided (Fig 1, *D*). No adverse events or recurrences occurred during the treatment or during the 12-month follow-up period.

## Discussion

Differentiating AGEP from GPP is challenging, particularly in patients without a history of psoriasis vulgaris, even with the Naranjo and European Study of Severe Cutaneous Adverse Reactions scoring system. In our case, the Naranjo score was 3/13, and the AGEP score was 7/12, indicating that the patient’s condition might be caused by macrolide antibiotics or traditional Chinese medicine, whereas the correlation is not significant. Additionally, this patient did not meet the European Rare and Severe Psoriasis Expert Network diagnostic criteria for GPP. However, glucocorticoid treatment failed.

Further genetic analysis revealed a pathogenic mutation in the IL36RN gene in our patient. Dysfunctional IL-36 receptor antagonists encoded by mutational IL36RN lead to the overactivation of IL-36 signaling, ultimately causing the inflammatory cascade.^2^ Currently, IL36RN mutations have been reported in AGEP.^3^ In addition, IL36RN gene mutations are found in most patients with GPP who do not exhibit psoriasis vulgaris and are related to the severity of GPP. These studies suggest that IL-36 signaling dysregulation is involved in the pathophysiology of both pustular diseases. The hypothesis that AGEP may be an aggravating form of GPP in patients with IL36RN positivity requires further investigation.

In the clinical setting, it is important to terminate the inflammatory cascade promptly to prevent the continuous progression of AGEP. Recent clinical trials have demonstrated the efficacy of spesolimab in IL36RN-positive patients with GPP.^4^ Based on these studies, we attempted to treat our patient with spesolimab. Successful clinical outcome confirmed the efficacy of spesolimab in the treatment of IL36RN-positive AGEP, which is insensitive to glucocorticoids.

## Conflicts of interest

None disclosed.
